# Promoting Consumers’ Sustainable Consumption of Online Retail Cold Chain Logistics Services: Extended Applications of SOR and Cognitive-Affective-Conative Theories

**DOI:** 10.3390/bs14090771

**Published:** 2024-09-02

**Authors:** Jiangmin Ding, Eon-Seong Lee

**Affiliations:** School of Business, Pusan National University, Busan 46241, Republic of Korea; djm0823@pusan.ac.kr

**Keywords:** online retail cold chain delivery services, consumer sustainable consumption, three-stage model, stimulus-organism-response (SOR) framework, cognitive-affective-conative (CAC) model

## Abstract

As food safety awareness rises and living standards improve, consumers have increasingly higher expectations for the efficiency and quality of cold chain logistics services. For cold chain logistics service providers, accurately understanding consumer psychology and enhancing their willingness to continue using the service—while guiding them to actively participate in green cold chain logistics practices—are necessary means to maintain competitiveness. Therefore, based on stimulus-organism-response and cognitive-affective-conative theories, this study constructs a three-stage model to explore the factors influencing consumers’ continuous consumption of online retail cold chain delivery services. This study substantiates that consumers’ intention to continue consuming proceeds through the following three stages: cognitive, affective, and conative. Specifically, the results indicate that consumer- and environment-oriented services significantly enhance consumer value and psychological empowerment, which further promotes their intention to continue using the service and participate in green practices. Moreover, psychological empowerment and perceived value fully and partially mediate the relationship between the two types of services and consumer sustainable consumption, respectively. These findings expand the research on cold chain consumption and deepen our understanding of how various factors influence consumer behavior.

## 1. Introduction

As consumption levels and quality of life improve, consumer demand for the quality of fresh food has increased. Moreover, in recent years, owing to factors such as the pandemic, a significant shift has occurred in consumer shopping behavior, with a notable increase in consumers ordering fresh food through online retail platforms [1]. This shift has amplified the importance of Business-to-Consumer (B2C) interactions in the fresh food sector. Temperature and time are the key factors that influence the quality of fresh food. Against this backdrop, cold chain logistics, characterized by low temperatures and rapid delivery, has become the primary distribution method for fresh products [2]. Compared with traditional logistics, consumers have higher expectations and requirements for cold chain logistics services in the B2C context [3]. Consequently, the online retail cold chain service industry faces increasingly higher pressure. With the enhancement in price transparency and delivery service efficiency, price and speed are no longer unique advantages for enterprises. Therefore, companies must seek new competitive advantages to enhance customer satisfaction [4]. In the fiercely competitive cold chain logistics market, achieving and maintaining excellent consumer value, and establishing and retaining a strong and loyal customer base, are essential conditions and operational goals for logistics service providers [5]. However, as sustainable development gains momentum and the public awareness of environmental protection strengthens, stakeholders are proposing novel requirements and expectations for environmental protection. Shi et al. [6] highlighted that cold chain logistics is a high carbon emission industry. Under various carbon regulations, incorporating carbon emissions into operational decisions and seeking effective approaches to reduce emissions and comply with restrictions are significant issues that should not be ignored in future cold chain logistics scheduling. Therefore, online retail cold chain service providers must strike a balance between profit and environmental protection, strengthen environmental initiatives to respond to green pressure, and fulfill the expectations of relevant stakeholders. Accordingly, Shi et al. [6] proposed an intelligent green scheduling system for cold chain logistics to support resource integration and coordination. However, existing studies tend to focus more on exploring relevant technologies and path optimization (e.g., [7,8]), with less attention paid to consumer participation and influence. However, consumers play an evident role as the users of these services. Achieving the two major operational goals of online retail cold chain service providers is inseparable from consumer participation. Therefore, relevant companies must conduct more detailed research from the perspective of consumers and further explore how to enhance consumer willingness to continue using the service and their green participation behavior.

Over the past few decades, within the context of a complex and ever-changing business environment, an increasing number of consumers have placed a strong demand on creating customer value. Consumer value is widely recognized as a key factor in organizational management, marketing strategies, and consumer behavior [9]. However, several scholars have underscored that, compared to basic customer satisfaction, further refinement and development of consumer value research are necessary (e.g., [10]), emphasizing that value is a crucial determinant of buyer satisfaction and repurchase decisions. Additionally, for consumers, a certain degree of psychological empowerment significantly enhances their willingness and actual behavior. Fuchs et al. [11] found that customers empowered to choose products to be sold exhibit a stronger demand for basic products. Consequently, numerous companies have become more proactive in involving their customers at various stages of the new product development process. Similarly, Lee et al. [12] confirmed that measures adopted by businesses to empower consumers strengthen their identification with the company, thereby positively influencing their perceived corporate social responsibility motives, attitudes toward the company, and purchase intentions. Moreover, consumers’ psychological behavior depends on their perceptions of products and services. If consumers have no awareness of a product or service, they will not develop any interest or desire for it and, consequently, will not make a purchase [13]. Today, consumers demand higher safety, quality, and convenience in food purchases. Owing to the perishability of fresh products, they have higher expectations for the timeliness and safety of cold chain delivery over general logistics. Lim et al. [5] highlighted that service quality and error handling are critical for consumer satisfaction with cold chain services. With increasing environmental awareness, consumers expect greener logistics. Diverse customer demands place greater pressure on delivery services, and poor performance can result in negative experiences, increased hidden costs, and customer loss. Hence, cold chain delivery companies must adopt flexible and adaptable strategies to fulfill multilevel consumer needs and realize their consumption value [5,14].

Therefore, this study focuses on the B2C online retail cold chain service industry. Based on the stimulus-organism-response (SOR) framework and cognitive-affective-conative (CAC) model, this study proposes a three-stage model to explore the factors that influence consumers’ sustainable consumption. This study aims to fill this gap in the understanding of consumer sustainable engagement in cold chain logistics by analyzing consumers’ psychological and actual behaviors. It seeks to provide insights for online retail cold chain service providers to achieve profits and environmental goals.

The remainder of this paper is organized as follows: In Section 2, we review the theoretical background and related research and thereby derive the hypotheses. Section 3 describes the research methodology. Section 4 presents the data analysis and results. Section 5 discusses the validation results; draws conclusions; and elucidates the academic and practical implications, limitations, and future research directions.

## 2. Theoretical Background and Hypotheses

### 2.1. Theoretical Background

The SOR model, pioneered in environmental psychology, was first introduced by environmental psychologists Mehrabian and Russell. It aims to explain and predict how different environmental stimuli affect human cognition, emotions, and behavior [15]. Scholars, such as Guo et al. [16], have utilized the SOR model to analyze the factors influencing consumers’ engagement and purchase intentions in e-commerce. Abbott et al. [17] used the model to explore the factors influencing impulsive online shopping behaviors among consumers. Lavuri et al. [18] explored the factors influencing consumers’ sustainable consumption behavior based on SOR theory. Based on this theory, Mansoor et al. [19] explored the interplay between consumer participation in sustainable consumption and social norms. However, sustainable consumption in logistics, particularly in cold chain logistics, has not been sufficiently studied. Therefore, this study applies the SOR framework to sustainable consumption of online cold chain delivery services, contributing to the enrichment of related research.

Additionally, consumer behavior typically comprises cognitive, affective, and conative aspects [20]. Cognitive aspects involve the development of consumers’ values, beliefs, and thoughts regarding an object or service (e.g., online retail cold chain services). Affective aspects encompass consumers’ feelings and attitudes toward objects. Conative aspects are related to consumers’ intentions and actual behavior toward an object [20,21]. To enhance our understanding of consumer psychology and behavior, this study utilizes the CAC model as another theoretical framework for investigation. Lavidge and Steiner [22] proposed this model for use in advertising research. Subsequently, it has been extended and widely applied to various fields, such as tourism [23], the use of mobile social networks [24], online shopping [21], and sustainable consumption [25].

Scholars have demonstrated that external and internal stimuli (S) affect an individual’s internal state (O), resulting in specific behavioral responses (R) [19]. The SOR model connects stimuli and responses through internal variables, emphasizing emotional and cognitive factors that align with some CAC theory viewpoints. According to CAC theory, consumer behavior encompasses cognition, affect, and consumption. The cognitive domain includes object-related awareness, knowledge, beliefs, thoughts, and attributes [25]. In this study, consumer- and environment-oriented services directly influence consumers’ perceptions of service quality and environmental friendliness. The affective domain represents likes, dislikes, and preferences, which form the emotional aspect of attitudes [25]. Similarly, in SOR theory, the organism is defined as an individual’s emotional state that triggers different consumer responses. Actual service perceptions and experiences provide immediate impressions to consumers. Consumer- and environment-oriented services help consumers fully understand the nature of a service. After experiencing a service, consumers exhibit various emotions, primarily perceived customer value and psychological empowerment. These emotions drive behavioral responses, such as continued consumption and green engagement. Therefore, these two types of services can be considered important factors influencing stimuli and cognition. Consumer research is a complex field, and integrating these two models establishes a solid theoretical foundation for this study, thereby enhancing our understanding of consumer psychology and behavior.

### 2.2. Hypotheses Development

First, consumers’ psychological behaviors during consumption are contingent on their perceptions of products and services. Aligned with two core operational objectives, online retail cold chain service providers pivot their services around consumer- and environment-centric approaches. From an enterprise perspective, consumer orientation implies the implementation of strategies centered on consumers [26], encompassing high-quality service delivery, efficient service performance, and comprehensive service initiatives. From a consumer perspective, consumer orientation denotes the extent to which consumers perceive the enterprise as centric to service delivery. Accordingly, Lee et al. [27] leveraged healthcare services as a focal point and discovered that service value and patient satisfaction are positively influenced by customer-oriented services. Thus, they emphasized that customer-oriented services are a core competency for a hospital’s sustainable development. Additionally, high-quality service delivery [28,29] and service innovation [30] oriented toward customers can enhance consumer satisfaction by creating customer value. Moreover, consumer orientation entails service providers’ understanding and swift response to consumer needs, striving to maximize consumer benefit. By actually utilizing relevant services, consumers can attain a degree of psychological empowerment. For example, consumers can autonomously and flexibly choose service providers, and select order and delivery times. This finding indicates that consumer autonomy is satisfied. Thus, we suggest that the following hypotheses:

**H1.**  *Customer-oriented services positively impact consumer perceived value.*

**H2.**  *Customer-oriented services positively impact consumer psychological empowerment.*

Furthermore, in the context of sustainable development, numerous businesses value environmentally oriented services equally. At the corporate level, environmental orientation refers primarily to various operational strategies implemented by companies with the aim of environmental conservation, including the use of green materials and eco-friendly advertising. Keszey [31] emphasized corporate environmental orientation as a significant driver of environmental marketing, while Zameer et al. [32] confirmed that environmental orientation significantly influences environmental performance. From the consumer perspective, environmental orientation implies the extent to which consumers perceive a company’s commitment to environmental protection (internal values and moral standards) as well as the recognition and responsiveness to environmental demands from its stakeholders [33]. By strengthening consumers’ awareness of environmental conservation, businesses are expected to provide more environmentally friendly services. After experiencing such services, consumers are more likely to personally realize the value of these services and perceive the company’s determination toward environmental protection. Consumers utilizing cold chain logistics services can choose whether to participate in green activities and, occasionally, even how to conduct these activities. They can choose to support companies that avoid excessive packaging or opt for eco-friendly packaging. Moreover, they can decide whether to participate in packaging recycling activities. These choices empower consumers with greater autonomy and foster a sense of responsibility. Therefore, we propose the following hypotheses:

**H3.**  *Environment-oriented services positively impact consumer perceived value.*

**H4.**  *Environment-oriented services positively impact consumer psychological empowerment.*

In recent years, various businesses have recognized the importance of enhancing consumer value, resulting in a surge in related research. However, in the service sector, research on consumer value remains somewhat fragmented [9]. Several marketing literatures discuss the concept of “value”, with scholars frequently referring to consumer value as “customer value”, “perceived value”, or “shopping value”. Watanabe et al. [34] surveyed 274 Brazilian organic food consumers and found that both functional and emotional values positively influence consumer trust. Furthermore, existing research has explored consumer value’s impact on purchase intentions in various domains, such as consumer food choices [34] and tourism [35,36], and the influence of emotional value on purchase intentions. Additionally, perceived value includes the awareness of social norms and environmental concerns, potentially influencing consumers’ green participation behavior. Danish et al. [37] surveyed Pakistani consumers and found that perceived value positively influences consumers’ choice of green electronic products. Moreover, studies have demonstrated a positive correlation between green perceived value and users’ green loyalty to public bicycles, indicating that consumers’ perceived value can guide more environmentally friendly behavior toward public bicycles [38]. Per the CAC model, consumer decisions begin with perceptions, thoughts, beliefs, and meanings regarding certain objects or issues [39]. Thus, the value perceived by consumers after experiencing the service induces their subsequent behavior. Therefore, we posit the following hypotheses:

**H5.**  *Customer perceived value positively impacts continuous use intention.*

**H6.**  *Customer perceived value positively impacts green engagement intention.*

Furthermore, we anticipate that consumer empowerment may positively impact consumers’ intentions for continued use and green participation. Specifically, empowerment provides consumers with more choices and autonomy. Fuchs et al. [11] found that customers empowered to choose among products tend to exhibit a stronger demand for basic product offerings. Therefore, actively involving customers in product development has become an innovative strategy for several businesses. Scholars have highlighted that measures adopted by businesses to enhance consumer capabilities strengthen consumers’ identification with a company, thereby positively influencing their perceived corporate social responsibility motives, attitudes toward the company, and purchase intentions [12]. Empowered consumers can freely choose among different service providers and decide whether to participate in green practices in cold chain logistics. In this case, they tend to exhibit behaviors conducive to environmental protection because of increased environmental awareness. Hence, we propose the following hypotheses:

**H7.**  *Consumer psychological empowerment significantly positively impacts continuous use intention.*

**H8.**  *Consumer psychological empowerment significantly positively impacts green engagement intention.*

**Figure 1:**
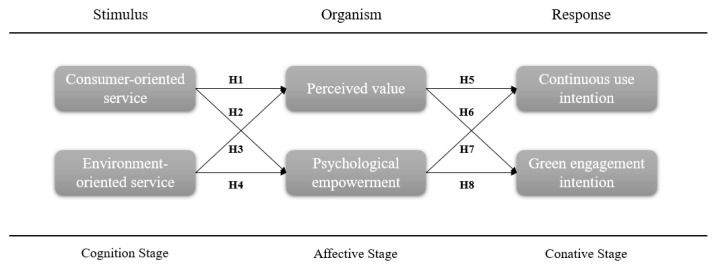
Research model.

Based on these arguments, this study devised a research model, as outlined in Figure 1.

### 2.3. Mediation Effects in the Three-Stage Model

Our proposed three-stage model posits that the sustainable consumption process for online cold chain delivery services involves the following three stages: service cognitive, affective, and conative (Figure 1). Specifically, consumer- and environment-oriented services can enhance consumer perceived value and psychological empowerment, which, in turn, increases their intentions for continued use and green engagement. This implies that perceived value and psychological empowerment act as mediators in this process. From the perspective of SOR and CAC theories, consumers experience emotions after being exposed to stimuli or cognition, which results in the formation of intentions and behaviors. As previously mentioned, consumer- and environment-oriented services help consumers gain a deeper understanding of the nature of services. By experiencing these services, consumer cognition is stimulated, enabling them to directly perceive service value and recognize the company’s consumer-centric approach and commitment to environmental protection. These diverse service experiences provide consumers with more knowledge regarding service usage, enabling them to choose providers more flexibly and decide independently whether and how to engage in green logistics. Perceived value’s mediating role in consumer behavior research has been validated multiple times. For example, Ullah [40] and Hapsari et al. [41] found that perceived value positively mediates the relationship between perceived service quality and satisfaction. Although psychological empowerment’s mediating role in consumer behavior research has been explored less frequently, existing studies have substantiated its effectiveness. For example, Han et al. [42] found that consumer psychological empowerment positively mediates the relationship between service fairness and satisfaction. Accordingly, to assess whether perceived value and psychological empowerment serve as mediators in the model, we identified key pathways in the model and conducted additional analyses.

## 3. Research Method

### 3.1. Sample and Data Collection

This study aims to verify the factors influencing consumers’ intentions for the sustainable consumption of cold chain logistics services through a quantitative analysis. To this end, we adapted several existing effective questionnaires to create an English questionnaire in the context of logistics services (e.g., [29,43,44]). To better align with the logistics field, we made some adjustments, such as modifying “service” to “logistics service” and further narrowing the scope of service providers to logistics service providers. Thereafter, the questionnaire was translated into Chinese and reviewed meticulously by experts in the field. Respondents were asked to rate the questionnaire items on a five-point Likert scale, with 1 indicating “strongly disagree” and 5 indicating “strongly agree”. To ensure the questionnaire’s precision and dependability, we administered a pilot survey to 20 randomly selected participants. Thereby, we identified minor issues within the questionnaire and, accordingly, made appropriate modifications and enhancements. 

Thereafter, questionnaires were distributed to various regions of China using the convenient sampling method. To enhance the collection efficiency, we employed a combination of offline and online distribution methods. Finally, we received 277 questionnaires. After screening out 14 questionnaires for missing values or insincere responses, we obtained 263 valid responses. Table 1 presents the respondents’ characteristics. The results indicate that the percentage of female respondents (61.22%) was higher than that of male respondents (38.78%). Among them, 62.74% were aged between 20 and 40 years, indicating that consumers in this age group use online services to purchase fresh food more frequently than other age groups. Overall, the regional distribution of respondents was relatively balanced, with the highest percentage in East China (35.74%) and the lowest in West China (11.03%). Additionally, respondents’ salary range was generally between CNY 3000 and CNY 12,000.

### 3.2. Variables Measurement

The research model consists of six constructs, including customer-oriented services (COS), environment-oriented services (EOS), perceived value (PV), psychological empowerment (PE), continuous use intention (CUI), and green engagement intention (GEI). Based on existing literature, we defined COS as the extent to which consumers perceive a company as implementing a consumer-centric service strategy (Lee et al. [27]); EOS as the degree to which consumers perceive a company’s commitment to environmental protection and its recognition and response to environmental demands from stakeholders (Keszey [31], Zameer et al. [32]); PV as the overall evaluation of subjective and objective factors of consumer experience [44]; PE as an individual’s subjective sense of being able to influence their environment (primarily referring to consumer agency in continuous use and green participation in the context of sustainable consumption in cold chain logistics); and CUI and GEI as consumer willingness to continue using the service and engage in green practices, respectively [29]. To ensure the validity of these constructs, we cited or modified all measurement items as much as possible from existing studies, and made further adjustments based on the context of online retail cold chain logistics services. Table 2 provides an overview of the measurement results for each variable.

## 4. Data Analysis and Results

### 4.1. Assessment of Measurement Model

First, we employed Harman’s single-factor analysis to confirm whether there is common method bias in this study [45]. The results revealed that six extracted factors had eigenvalues greater than 1.0 were extracted, accounting for approximately 74.843% of the total variance. The first extracted factor explained 15.044%, which was less than 50% of the total variance. Overall, there is no significant common method bias in this research. We further assessed the issue of multicollinearity by testing the variance inflation factor (VIF) of the variables. The results showed that the VIF values for the sample were below the critical threshold of 3.3 [46], ranging from 1.709 to 2.276. Therefore, there are no multicollinearity issues present in the sample used in this study.

Subsequently, we conducted tests for reliability and validity, and further validation was performed using structural equation modeling (SEM). Specifically, we conducted a confirmatory factor analysis to verify the reliability and validity of the measurement variables in the model. To assess reliability, we used Cronbach’s α, which reflects internal consistency, as well as composite reliability (CR). CR considers factor loadings of measurement variables, measurement error, and average variance extracted (AVE), representing the average explanatory power of the measurement variables for the latent variables. As shown in Table 2, the reliability analysis meets the acceptable standards, with Cronbach’s α exceeding 0.7, AVE exceeding 0.5, and CR exceeding 0.7, confirming the reliability of the measurement variables for each construct [47].

Furthermore, we evaluated convergent and discriminant validity. Convergent validity indicates the extent to which the variables measuring each construct converge on that construct, while discriminant validity measures the distinction between a specific construct and other constructs. As shown in Table 2, the analysis of convergent validity demonstrates that the standardized estimates of the measurement variables for each construct exceed the 0.5 threshold, confirming convergent validity. The analysis of discriminant validity (Table 3) reveals that the square roots of the AVEs for each construct are greater than the correlations between constructs, thereby ensuring discriminant validity [48]. However, some scholars have pointed out that the Fornell–Larcker criterion is insufficient for detecting discriminant validity issues in general research contexts [48]. Therefore, Henseler et al. [49] recommended an alternative method for assessing discriminant validity called the Heterotrait–Monotrait (HTMT). They also demonstrated the effectiveness of HTMT through Monte Carlo simulations. Given this robust technique, our study also employed the HTMT method to test discriminant validity. The heuristic for HTMT testing is that if the HTMT value exceeds 0.85 or 0.90 [47,49], it indicates a potential issue with discriminant validity. The results of the HTMT test, as shown in Table 3, meet the criteria for both HTMT 0.85 and HTMT 0.90. This indicates that the measurement model possesses adequate validity and discriminant validity.

### 4.2. Hypothesis Test

In our study, a structural equation model was constructed for hypothesis testing. The R^2^ values for perceived value, psychological empowerment, continuance intention, and green engagement intention were 0.218, 0.133, 0.214, and 0.154, respectively, meeting the significance standard of R^2^ ≥ 0.1 as suggested by Falk and Miller [50]. Additionally, the fit indices were as follows: χ^2^/df = 1.576 (<3), GFI = 0.915 (>0.9), RMSEA = 0.047 (<0.1), CFI = 0.964 (>0.9), NFI = 0.909 (>0.9), and NNFI = 0.957 (>0.9). These indicators all met the established criteria, suggesting that the model fit of our study is acceptable.

**Figure 2:**
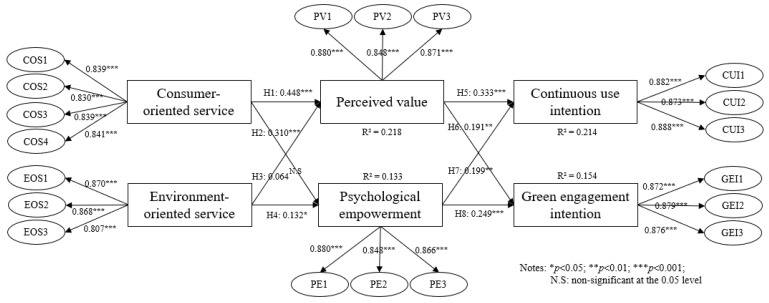
PLS structural model results.

Then, we used bootstrapping, a technique that involves repeated sampling to evaluate statistics, with 5000 iterations to test the veracity of the research hypotheses. Noteworthily, no consensus exists regarding the optimal number of bootstrap samples to generate; however, a larger value is generally considered better. According to Preacher and Hayes’ recommendations [51], at least 5000 resamples should be used in the final report, which helps minimize the estimates’ sampling variance, resulting in a relatively accurate sampling distribution. Therefore, 5000 iterations are commonly used in typical studies. Figure 2 and Table 4 present the hypothesis test and results.

H1 and H2 predict that consumer-oriented services will have a significant impact on consumer value and psychological empowerment. The analysis results show that the β values are 0.448 and 0.31, with *p*-values both less than 0.001, thus supporting H1 and H2. However, for H3, the β value is 0.064 and the *p*-value is 0.24, which is greater than 0.05. Therefore, H3 is rejected, indicating that the impact of environment-oriented services on consumer perceived value was not verified in this study. Conversely, the results for H4 show a β value of 0.132 and a *p*-value less than 0.05, thus supporting H4. Additionally, H5 and H6 predict that consumer perceived value will positively influence their willingness to continue using the service and to engage in green practices. The results show β values of 0.333 and 0.199, with *p*-values both less than 0.01, thus supporting H5 and H6. Similarly, H7 and H8 predict that psychological empowerment will positively affect the willingness to continue using the service and engage in green practices. The results show β values of 0.191 and 0.249, with *p*-values both less than 0.01, thus supporting H7 and H8.

### 4.3. Mediation Effect Test

We further explored the indirect effects between variables through mediation analysis, as shown in Table 5. We performed bootstrapping with 5000 resamples and used bias-corrected confidence intervals. The results indicated that perceived value exhibited a positive mediating effect on the relationship between consumer-oriented services and sustainable consumer behaviors (continued use and green participation), with β values of 0.149 ([0.092–0.212]) and 0.089 ([0.039–0.147]), respectively, both significant at *p* < 0.01. By contrast, the mediation analysis of perceived value between environmentally oriented services and sustainable consumption revealed β values of 0.021 ([−0.006–0.0.057]) and 0.013 ([−0.004–0.0.036]), with *p*-values greater than 0.05, indicating that perceived value did not exhibit a significant mediating effect in this context. However, psychological empowerment’s mediating role was supported, with β values of 0.059, 0.077, 0.025, and 0.033 (all confidence intervals do not include 0), all significant at *p* < 0.05. Thus, psychological empowerment exhibited a full mediating effect. The results of the total indirect effect analysis also show that four paths have been validated. Overall, perceived value served as a partial mediator between service perception and sustainable consumption, whereas psychological empowerment acted as a full mediator. This aligns with our proposed three-stage model, which posits that the sustainable consumption process for online cold chain delivery services involves the following three stages: cognitive, affective, and conative.

## 5. Discussion and Conclusions

Cold chain logistics plays a crucial role in maintaining a global supply of fresh food. With improvements in living standards and purchasing power, consumers have higher expectations of cold chain logistics services. Therefore, accurately understanding consumer psychology and adopting precise strategies to enhance consumer willingness for continued usage are important challenges for businesses. Moreover, guiding consumers to participate in green practices in cold chain logistics under environmental pressure is an important strategic goal for enterprises. Therefore, drawing on the SOR and CAC model, this study introduces a three-stage model aimed at investigating the factors influencing consumers’ continuous consumption of online retail cold chain logistics delivery services. Based on this, we tested our hypotheses by surveying 263 Chinese consumers who had experience in purchasing fresh food online (i.e., had used cold chain delivery services).

First, H1 and H2, which posit that consumer-oriented services enhance consumer perceived value and psychological empowerment, were supported. This finding is consistent with Lee et al.’s results [27]. Owing to the direct impact of cold chain logistics on the quality and safety of fresh products, efficient and high-quality services are more likely to fulfill the expectations of modern consumers. Additionally, consumer-oriented services provide consumers with a greater sense of choice and autonomy, thereby enhancing psychological empowerment.

However, contrary to our expectations, environment-oriented services did not significantly enhance consumer perceived value, as hypothesized. Interestingly, this effect was not observed in the present study. Instead, environment-oriented services enhanced consumer psychological empowerment. The likely reason for this is that consumers perceive value from consumer-oriented services more directly, whereas environment-oriented services tend to serve educational and promotional purposes. Consequently, consumers may develop a sense of responsibility and confidence when choosing different suppliers and engaging in green initiatives. In the context of cold chain logistics, consumer-centered services place greater emphasis on service accuracy and timeliness, which are critical to consumers. Therefore, such services directly increase consumer perceived value. By contrast, while the greening of cold chain logistics (environment-oriented services) improves consumers’ awareness of green cold chain services and enhances their sense of responsibility and their subjective initiative to decide whether and how to participate in green logistics, it does not invariably convey value to consumers directly, clearly, and perceptibly. Thus, this should be a strategic focus for cold chain logistics service providers. Numerous studies have substantiated the importance of technological improvements and other measures for reducing costs while implementing green cold chain logistics (e.g., [7,8]). However, as the main consumers of B2C cold chain delivery services, sustainable consumption should be a significant area of focus. Exploring how consumers can be engaged in this process and how to enhance perceived value when offering environment-oriented services warrants further investigation.

Furthermore, this study’s findings support H5 and H6, indicating that perceived value enhances consumer willingness to continue using and engaging in green practices in cold chain logistics, implying that perceived value can promote consumer sustainable consumption of online retail cold chain logistics. This aligns with the findings of Danish et al. [37] and Chen [38], who suggest that value as a critical product attribute remains significant for consumers and is a key reason for their loyalty. Moreover, with increasing environmental awareness, consumers’ perceived value includes their understanding of social norms and environmental issues, which further influences their green engagement behavior. Finally, the positive effect of consumers’ psychological empowerment on their continued use and green participation intention is supported.

Moreover, the positive effect of consumer psychological empowerment on their continued use and green engagement intentions is supported. It is well known that consumers face varied choices, and whether they continue using the same platform’s services or participate in green practices depends on the consumers themselves. Empowered consumers with psychological authorization have greater autonomy; therefore, they frequently exhibit more proactive responses and actual behaviors. Scholars have highlighted that measures adopted by companies to enhance consumer capabilities contribute to strengthening consumers’ identification with the company, thereby positively impacting their perception of the company’s corporate social responsibility motives, attitudes toward the company, and purchase intentions [12]. Finally, the mediation analysis results indicate that consumer psychological empowerment and perceived value fully and partially mediate the relationship between the two types of services and sustainable consumer behavior, respectively. This is consistent with the three stages of our proposed model, which posits that the sustainable consumption process for consumers’ online cold chain delivery services comprises cognitive, affective, and conative stages.

Based on these findings, this study makes the following contributions: From an academic standpoint, first, this study extends SOR and CAC theories’ application to the field of online retail cold chain logistics services, which not only validates these two theories again but also enriches the related research on cold chain logistics. Second, this study establishes a three-stage model based on the SOR framework, confirming that consumers’ intention to continuously consume online retail cold chain logistics services undergoes the following three stages: cognitive, affective, and conative. Third, this study focuses on consumers’ intrinsic psychological factors, including the exact impact of perceived value and psychological empowerment, thus filling a gap in the related research in the field of cold chain services. This study’s findings will expand the study of customer value and psychological factors in the context of cold chain consumption, deepening our understanding of how various factors influence user behavior.

From a practical standpoint, this study provides valuable guidance for cold chain logistics service providers and related businesses. First, companies can strategically improve their services and products to enhance consumer trust and loyalty by gaining a deeper understanding of consumer perceptions of different service orientations. Second, this study offers insights for cold chain logistics service providers to formulate service strategies and innovation plans. Companies can plan and implement innovative measures by clearly understanding consumer dual demands for service quality and environmental friendliness. Moreover, encouraging consumer participation in green cold chain logistics practices can help reduce production costs and create a competitive advantage. Finally, this study validated the significant impact of consumer value and psychological empowerment. This suggests that service value as a fundamental attribute has invariably been valued by consumers, and modern consumers prefer to be respected and empowered rather than led by companies. Thus, firms can implement different marketing strategies based on consumer psychology.

This study has some limitations. First, despite our best efforts, a risk of bias still exists concerning the survey data’s accuracy. Second, institutional, cultural, and economic differences exist among countries and regions, which may precipitate variations in consumer behavior. For example, developed countries typically have more advanced technologies and environmental policies than developing countries, making comparative studies valuable. Cold chain delivery requires high standards of both timeliness and safety. However, owing to differences in area, economic level, infrastructure, delivery methods, and time can vary between countries and regions. Even within a single country, such as China, disparities in the demand for fresh foods, such as seafood, and variations in road conditions between coastal and inland areas, can result in differences in delivery practices. Therefore, in the future, we will use a larger sample size to examine cross-regional and cross-cultural differences more comprehensively.

## Figures and Tables

**Table 1:** Characteristics of respondents (N = 263).

Category	Items	Frequency	Percentage (%)
Gender	Male	102	38.78%
	Female	161	61.22%
Age	Less than 20	41	15.59%
	20–40	165	62.74%
	41–60	47	17.87%
	More than 60	10	3.80%
Educational Background	High school and below	32	12.17%
	Bachelor’s degree (including college)	186	70.72%
	Master’s degree and above	45	17.11%
Careers	Student	78	29.66%
	Self-employed individual	35	13.31%
	Employee	129	49.05%
	Others	21	7.98%
Regions	Eastern China	94	35.74%
	Southern China	47	17.87%
	Western China	29	11.03%
	Northern C Prof. Dr. Rogelio Puente-Diazhina	48	18.25%
	Central China	45	17.11%
Incomes (CNY)	Less than 3000	21	7.98%
	3000–6000	57	21.67%
	6001–9000	99	37.64%
	9001–12,000	54	20.53%
	More than 12,000	32	12.17%

**Table 2:** Reliability and validity test of measurement.

Variables and Measurement	Loading
Customer-oriented service (α = 0.858, C.R. = 0.860, AVE = 0.701).	
COS1. Logistics service providers should understand the needs of consumers.	0.839
COS2. Logistics service providers should respond quickly to consumer needs.	0.830
COS3. Logistics service providers should endeavor to maximize benefits for consumers.	0.839
COS4. Logistics service providers should design and launch products and services with the consumer in mind.	0.841
Environment-oriented service (α = 0.808, C.R. = 0.828, AVE = 0.721).	
EOS1. Firms have a responsibility to protect the environment.	0.870
EOS2. Firms and their employees understand the importance of protecting the environment.	0.868
EOS3. Firms should have a clear policy for developing and implementing environmental management strategies.	0.807
Perceived value (α = 0.835, C.R. = 0.840, AVE = 0.751).	
PV1. Product delivery was accurate and satisfactory.	0.880
PV2. The delivery person was friendly and the service was satisfactory.	0.848
PV3. The services provided reflect the service provider’s concern and commitment to environmental protection.	0.871
Psychological empowerment (α = 0.831, C.R. = 0.832, AVE = 0.747).	
PE1. I am free to choose my shopping platform and service provider.	0.880
PE2. I am free to choose whether or not to use reusable packaging.	0.848
PE3. I am free to choose whether or not to participate in the recycling of transport packaging.	0.866
Continuous use intention (α = 0.856, C.R. = 0.858, AVE = 0.776).	
CUI1. I will prioritize this platform and logistics provider for future purchases.	0.882
CUI2. If all other attributes (price, product, quality, etc.) are similar, I will continue to purchase products from that platform.	0.873
CUI3. I would recommend the platform to my family and friends in the future.	0.888
Green engagement intention (α = 0.848, C.R. = 0.851, AVE = 0.767).	
GEI1. I like to simplify packaging when ordering and delivering products.	0.872
GEI2. I would like to use reusable bags or boxes.	0.879
GEI3. I am willing to cooperate with other environmental protection strategies of the company and contribute to environmental protection.	0.876

**Table 3:** Analysis of discriminant validity.

	Construct	COS	CUI	EOS	GEI	PE	PV
AVE	COS	0.837					
	CUI	0.448	0.881				
	EOS	0.244	0.273	0.849			
	GEI	0.374	0.375	0.146	0.876		
	PE	0.342	0.366	0.207	0.354	0.865	
	PV	0.463	0.434	0.173	0.330	0.526	0.867
HTMT	COS						
	CUI	0.522					
	EOS	0.293	0.329				
	GEI	0.440	0.439	0.170			
	PE	0.405	0.433	0.247	0.418		
	PV	0.543	0.508	0.206	0.390	0.630	

Notes: COS = customer-oriented service; EOS = environment-oriented service; PV = perceived value; PE = psychological empowerment; CUI = continuous use intention; GEI = green engagement intention. Square root values of AVEs are shown on the diagonal.

**Table 4:** Hypothesis testing and results.

Hypothesis	Path	Path Coefficient	*p* Value	Results
H1	COS -> PV	0.448	<0.001	Supported
H2	COS -> PE	0.31	<0.001	Supported
H3	EOS -> PV	0.064	0.12	Not Supported
H4	EOS -> PE	0.132	0.014	Supported
H5	PV -> CUI	0.333	<0.001	Supported
H6	PV -> GEI	0.199	0.001	Supported
H7	PE -> CUI	0.191	0.001	Supported
H8	PE -> GEI	0.249	<0.001	Supported

Notes: COS = customer-oriented service; EOS = environment-oriented service; PV = perceived value; PE = psychological empowerment; CUI = continuous use intention; GEI = green engagement intention.

**Table 5:** Mediation analysis results.

Effect	Path	Estimate	SE	LL	UL
Total indirect effect	COS -> CUI	0.208	0.038 ***	0.15	0.274
	COS -> GEI	0.166	0.037 ***	0.109	0.232
	EOS -> CUI	0.047	0.026 *	0.008	0.093
	EOS -> GEI	0.046	0.022 *	0.012	0.085
Specific indirect effect	COS -> PV -> CUI	0.149	0.037 ***	0.092	0.212
	COS -> PV -> GEI	0.089	0.033 **	0.039	0.147
	EOS -> PV -> CUI	0.021	0.019	−0.006	0.057
	EOS -> PV -> GEI	0.013	0.012	−0.004	0.036
	COS -> PE -> CUI	0.059	0.025 **	0.024	0.104
	COS -> PE -> GEI	0.077	0.029 **	0.034	0.13
	EOS -> PE -> CUI	0.025	0.015 *	0.005	0.054
	EOS -> PE -> GEI	0.033	0.018 *	0.008	0.067

Notes: LL and UL represent the lower limit and upper limit of the bootstrapping 95% confidence intervals, respectively; * *p* < 0.05; ** *p* < 0.01; *** *p* < 0.001.

## Data Availability

The original contributions presented in the study are included in the article, further inquiries can be directed to the corresponding author.
